# Prophylactic negative pressure wound therapy reduces surgical site infection in gastrointestinal, hepatobiliary, and pancreatic laparotomy: A systematic review and meta-analysis

**DOI:** 10.1097/MD.0000000000050174

**Published:** 2026-08-14

**Authors:** Yanyun Long, Xiaohui Gu, Xin Wang, Kelei Wang, Yong Guan, Shaocong Wang, Chao Liu

**Affiliations:** a Department of General Surgery, The Second Affiliated Hospital of Zhengzhou University, Zhengzhou, Henan, China; b Department of Hepatobiliary and Pancreatic Surgery, The Fifth Affiliated Hospital of Zhengzhou University, Zhengzhou, Henan, China; c Department of Emergency, The Fifth Affiliated Hospital of Zhengzhou University, Zhengzhou, Henan, China.

**Keywords:** gastrointestinal surgery, hepatobiliary surgery, meta-analysis, negative pressure wound therapy, pancreaticoduodenectomy, surgical site infection, systematic review

## Abstract

**Background:**

Surgical site infections (SSIs) remain a major complication following gastrointestinal, hepatobiliary-pancreatic, and pancreatic surgery. This systematic review and meta-analysis aimed to assess the efficacy of prophylactic negative pressure wound therapy (NPWT) in reducing SSIs following closed abdominal incisions.

**Methods:**

A systematic search was conducted in PubMed, Embase, the Web of Science, and the Cochrane Library for studies published from January 2016 to December 2025; all databases were last searched on December 31, 2025, without language restrictions. Both randomized and nonrandomized studies were included. The primary outcome was SSI, while wound dehiscence (WD) was considered a secondary outcome. The risk of bias was assessed using the Cochrane Risk of Bias Tool 2.0 (Cochrane, London, UK). Meta-analyses were performed using Stata/MP version 18.0 (StataCorp LLC, College Station) with a random-effects model and Hartung-Knapp-Sidik-Jonkman correction. A Sankey diagram was generated using Origin 2022 (OriginLab Corporation, Northampton).

**Results:**

A total of 10 studies (6 randomized trials and 4 retrospective studies) involving 3301 patients were included. Among them, 900 patients received NPWT, and 2401 patients received standard dressings. A random-effects model with Hartung-Knapp-Sidik-Jonkman correction meta-analysis indicated that NPWT significantly reduced the overall incidence of SSI compared with standard dressings (odds ratio [OR] = 0.63; 95% confidence interval [CI] = 0.41–0.99; *P* = .04). NPWT also significantly decreased the rates of superficial SSI (OR = 0.51; 95% CI = 0.37–0.69; *P* < .01) and deep SSI (OR = 0.45; 95% CI = 0.23–0.87; *P* = .03). However, no significant differences were observed for organ-space SSI or WD.

**Conclusion:**

Prophylactic NPWT reduces the risk of SSI among patients undergoing gastrointestinal, hepatobiliary-pancreatic, and pancreatic abdominal surgeries. Nevertheless, the rates of organ-space SSI and WD are comparable to those associated with standard dressings.

## 1. Introduction

Postoperative wound complications, including surgical site infection (SSI), wound dehiscence (WD), and the formation of hematomas or seromas, are frequently encountered following abdominal surgery. SSIs not only extend the length of hospital stay but also impede or undermine adjuvant therapy and elevate patient morbidity and mortality.^[1]^ Several perioperative risk factors have been recognized as predictors of SSI, including contaminated or dirty wounds, high body mass index, smoking, intraoperative oxygen fraction, and hypoalbuminemia.^[2]^ In gastrointestinal (GI) surgeries, the risk of SSI is notably high owing to the presence of abundant gut microbiota and inevitable contact with intestinal contents, which generally classifies these procedures as clean-contaminated.^[3]^ In hepatobiliary and pancreatic surgeries, where the surgical sites may also be clean-contaminated or contaminated, the risk is further heightened.^[4]^

Conventional perioperative measures, including preoperative antibiotic prophylaxis, aseptic surgical techniques, maintenance of normothermia, and optimization of patient risk factors, have been adopted to mitigate the risk of SSIs.^[5,6]^ Nevertheless, these interventions alone cannot ensure the prevention of SSIs.^[7]^ Negative pressure wound therapy (NPWT) has been advocated by multidisciplinary international panels as a potential supplementary approach for SSI prevention.^[8]^ NPWT applies continuous negative pressure, generally ranging from −80 to −125 mm Hg, to the wound via a vacuum device. This actively eliminates wound exudate, reduces dead space, alleviates tissue edema, and promotes the formation of granulation tissue. Commercially available systems include traditional vacuum-assisted closure and newer portable devices, such as PREVENA (an incision management system) and PICO (a negative pressure wound therapy system).^[9]^ Previous meta-analyses indicate that incisional NPWT reduces the risk of SSIs compared with standard dressings, with evidence of moderate to high quality.

In the past decade, a multitude of studies have compared NPWT with conventional wound care in abdominal surgery. Some studies report substantial reductions in the incidence of SSI,^[10]^ whereas others observe no significant disparity between NPWT and standard dressings.^[11]^ Considering the growing number of studies in this domain,^[12]^ an updated systematic review and meta-analysis is necessary to more precisely evaluate the efficacy of NPWT in preventing SSI among high-risk patients undergoing GI, hepatobiliary-pancreatic (HBP), and pancreatic surgery. This study intends to elucidate the role of prophylactic NPWT in reducing the incidence of SSI after abdominal surgery.

## 2. Methodology

This systematic review and meta-analysis were carried out in accordance with the Preferred Reporting Items for Systematic Reviews and Meta-Analyses 2020 statement.^[13]^ The study protocol was prospectively registered in the International Prospective Register of Systematic Reviews (registration No. CRD420261451847). Two independent reviewers (Yanyun Long and Xiaohui Gu) executed all screening and data extraction procedures. Disagreements were addressed through discussion or, when required, consultation with a senior author (Chao Liu).

### 2.1. Search strategy

Comprehensive literature searches were carried out using Medical Subject Headings (MeSH) terms and title/abstract keywords associated with surgical procedures and NPWT. The targeted surgical operations included gastrectomy, rectal resection, colectomy, hepatectomy, liver transplantation, cholecystectomy, pancreatectomy, and pancreatoduodenectomy. NPWT-related terms included “Negative Pressure Wound Therapy,” “Vacuum-Assisted Closure,” “Negative Pressure,” “NPWT,” and the corresponding MeSH headings. Searches were conducted across PubMed, Embase, Web of Science, and the Cochrane Library for publications spanning from January 2016 to December 2025; all databases were last searched on December 31, 2025, without any language restrictions. (In the Scopus database, only 2 potentially relevant records were identified, and they were not merged with the records from the other 4 databases.) In PubMed, Boolean operators (AND/OR) were applied to combine MeSH terms and free-text keywords to enhance sensitivity to the greatest extent. The search results were exported in Research Information Systems format and managed using EndNote 2022 (Clarivate, Philadelphia). After merging the databases, duplicate records were eliminated. The titles and abstracts were independently screened by 2 reviewers (Yanyun Long and Xiaohui Gu), and additional manual checks of the reference lists were conducted to identify further eligible studies.

### 2.2. Eligibility

Based on the PICOS framework (Participants, Intervention, Comparison, Outcomes, Study Design),^[14]^ studies that directly compared NPWT with conventional dressings for closed abdominal incisions following GI, HBP, or pancreatic surgery were included. Eligible study designs included randomized controlled trials (RCTs) as well as prospective or retrospective comparative cohort studies. The inclusion criteria were:

1. Adult patients who underwent GI, HBP resection, or pancreatic resection via laparotomy (either elective or emergency).
2. Immediate intraoperative application of commercial or simplified NPWT systems for primarily closed incisions.
3. Prophylactic NPWT as an intervention, with control groups receiving conventional postoperative dressings (e.g., iodine or sterile gauze).
4. Reporting of at least 1 outcome within 30 days: SSI (including superficial, deep, or organ-space), WD, length of hospital stay, wound healing time, or dressing-related costs.

The exclusion criteria were pediatric studies, case reports, single-arm studies, studies lacking extractable outcome data, animal studies, reviews, editorials, or studies in which wound healing was notably influenced by comorbidities or stomas.

### 2.3. Outcome definitions

SSI was categorized in accordance with the Centers for Disease Control and Prevention criteria into superficial SSI (sSSI), deep SSI (dSSI), or organ-space SSI. WD was defined as any spontaneous separation of the skin or fascia. The primary endpoint was incisional SSI; secondary endpoints encompassed sSSI, dSSI, organ-space SSI, and WD.

### 2.4. Data extraction and quality assessment

Data were independently extracted by 2 reviewers (Yanyun Long and Xiaohui Gu) via a standardized pilot form. The extracted variables included the first author, the publication year, the country of origin, the study design, the sample size, the patient population, the type of NPWT device, the applied pressure, the treatment duration, the control treatment, the definitions of SSI, the timing of outcome assessment, the number of patients in each group, and the number of SSI events in each group. RCTs were assessed using the Cochrane Risk of Bias Tool 2.0, which evaluates randomization, deviations from the intended interventions, missing data, outcome measurement, and selective reporting. Cohort studies were assessed using the Newcastle-Ottawa Scale (NOS).

### 2.5. Statistical analysis

For dichotomous outcomes, odds ratios (ORs) with 95% confidence intervals (CIs) were calculated. Considering the limited number of studies available for several outcomes and the potential between-study variability, all meta-analyses were performed using a random-effects model with restricted maximum likelihood (REML) estimation. The Hartung-Knapp-Sidik-Jonkman (HKSJ) adjustment was applied to obtain more conservative CIs.

Statistical heterogeneity was assessed using the Cochran *Q* test, *I*^2^ statistic, and τ^2^. *I*^2^ values of 25%, 50%, and 75% were considered to represent low, moderate, and high heterogeneity, respectively.

To assess the robustness of the pooled estimates, leave-one-out sensitivity analyses were conducted by sequentially excluding each included study (please refer to the supplementary material for further details). Particular attention was given to outcomes in which a single study contributed a high proportion of the total weight, especially the analyses presented in Figures 4 and 5. Leave-one-out sensitivity analyses are shown in Figures S1 to S5, Supplemental Digital Content 1, and a summary of the sensitivity analyses is provided in Table S1, Supplemental Digital Content 2.

**Figure 1.**
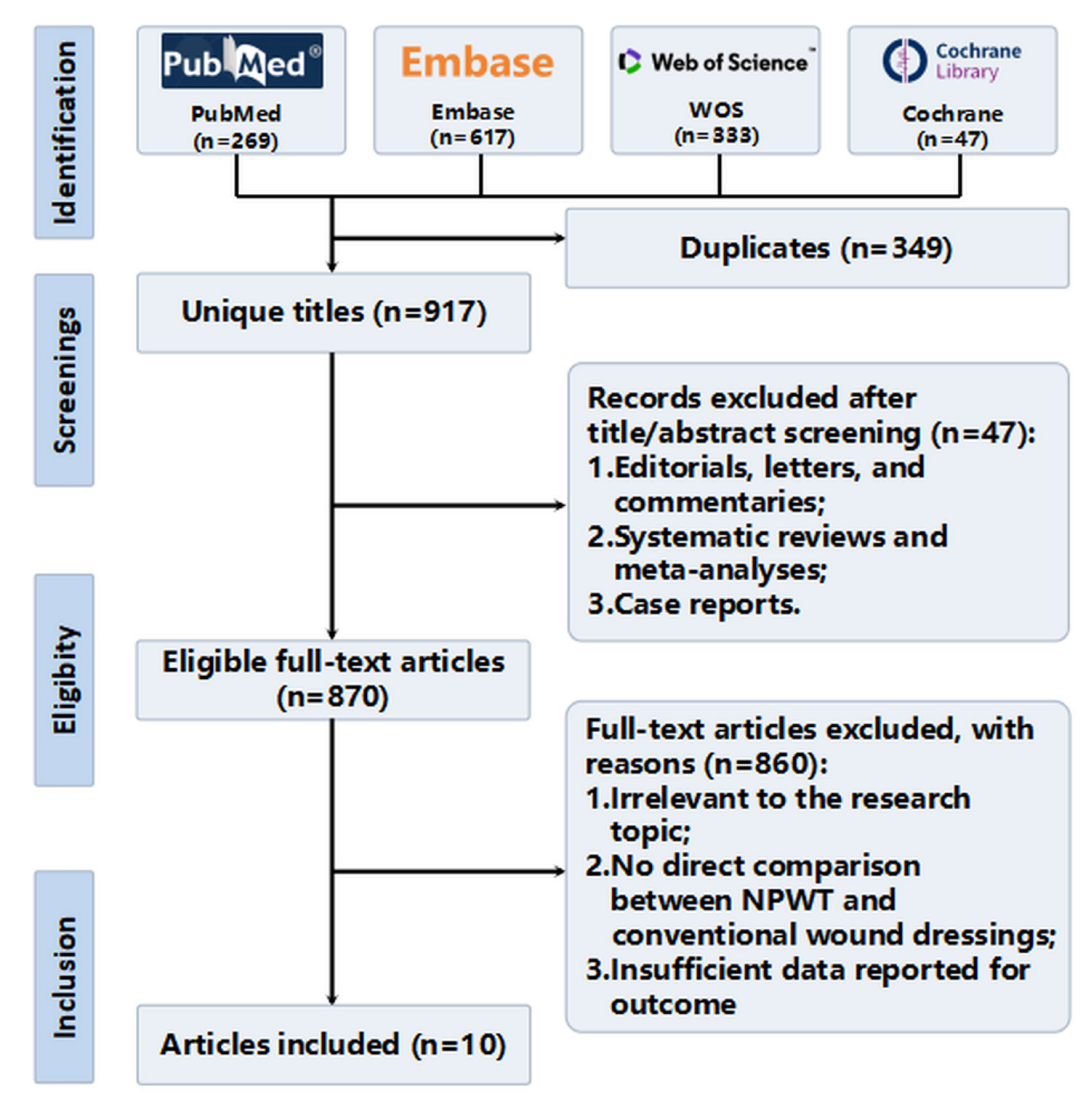
PRISMA flow diagram of study selection. NPWT = negative pressure wound therapy, PRISMA = Preferred Reporting Items for Systematic Reviews and Meta-Analyses.

**Figure 2.**
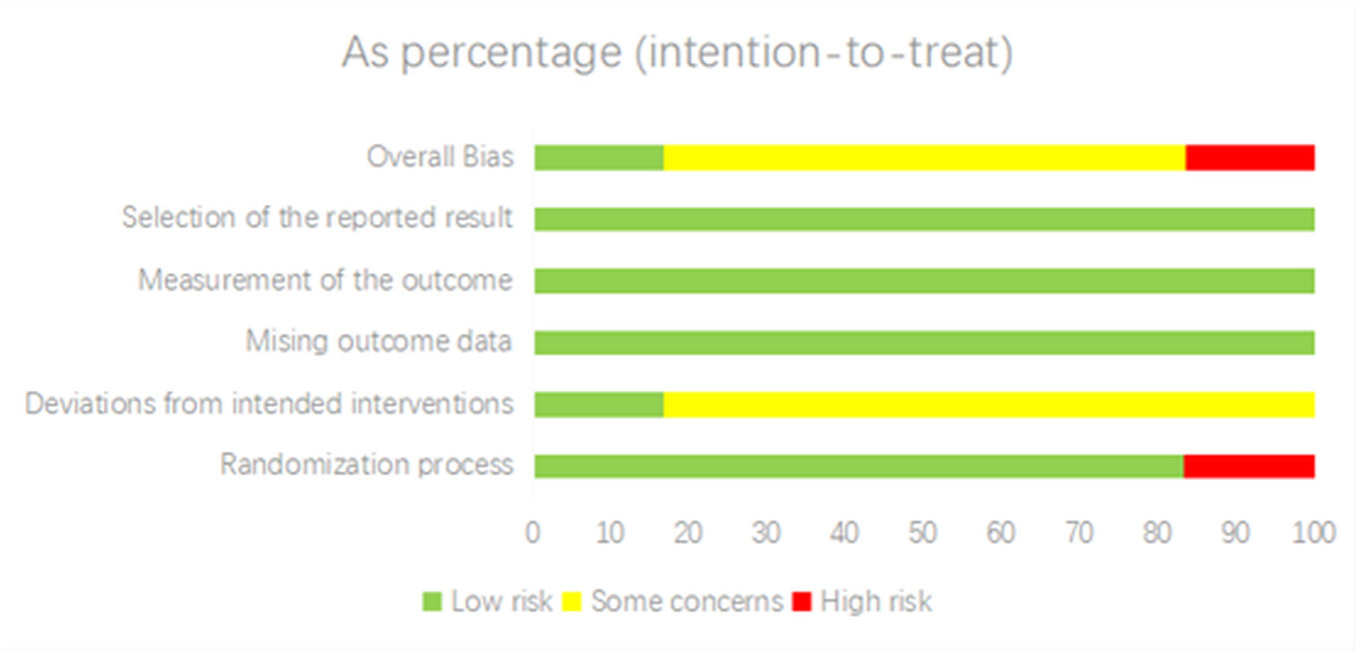
Summary of risk of bias in 6 RCT studies. RCT = randomized controlled trial.

**Figure 3.**
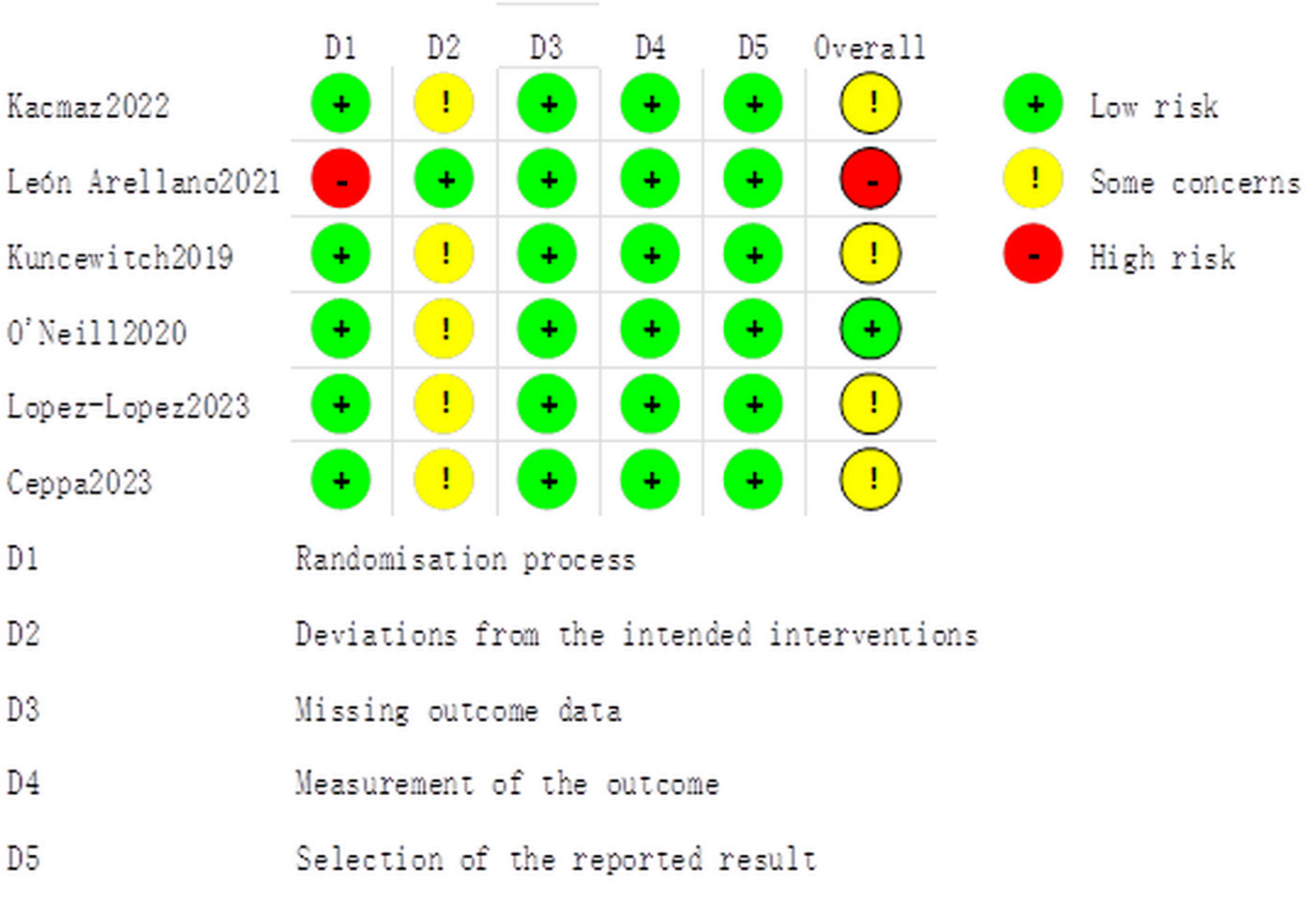
Risk of bias assessment opinions for 6 RCT studies. RCT = randomized controlled trial.

## 3. Results

### 3.1. Study selection

The literature screening process is presented in Figure 1. The initial search retrieved 1266 records from 4 databases. After eliminating 349 duplicate records, 917 records were screened by title and abstract, of which 47 were excluded. The remaining 870 reports were assessed for eligibility, and 860 were excluded due to irrelevance after full-text review. Finally, 10 eligible studies (6 RCTs^[11,15–19]^ and 4 retrospective studies^[12,20–22]^) involving 3301 patients, with 900 in the NPWT group and 2401 in the control group, were included.

### 3.2. Study characteristics

The included studies covered a diverse range of abdominal, GI, and hepatopancreatobiliary procedures. Seven studies were conducted in the United States,^[11,12,15,17,19,20,22]^ 2 in Spain,^[16,18]^ and 1 in Canada.^[21]^ Five studies involved gastrectomy or colorectal surgery,^[12,15,16,19,22]^ and 5 involved hepatobiliary or pancreatic surgery.^[11,17,18,20,21]^ The NPWT devices evaluated included PREVENA (3 studies^[16,19,21]^), PICO (3 studies^[11,15,18]^), and other NPWT instruments (4 studies^[12,17,20,22]^). The applied pressure ranged from −80 mm Hg to −125 mm Hg, with treatment durations of 2 to 7 days (Table 1).

**Table 1:** Characteristics of included studies evaluating negative pressure wound therapy (NPWT) for surgical site infection (SSI) prevention.

Source/country/study type	No. of patients NPWT/control groups	Outcome	Details of NPWT	Type of procedure	Antibiotic therapy	Other preoperative preparations	Follow-up
Kacmaz et al^[15]^/USA/RCT	24/26	SWC (SSI, hematoma, seroma, and wound dehiscence, 30 d)	PICO (setting, −80 mm Hg for 7 d)	Open colorectal cancer (CRC) surgery	All patients received ampicillin and sulbactam.	Hair removal	30 d
León Arellano et al^[16]^/Spain/RCT	75/73	SSI (7, 15, and 30 d)	PREVENA	Open surgery of the colon and/or rectum	All patients received.	Not mentioned	30 d
Kuncewitch et al^[17]^/USA/RCT	36/37	sSSI, dSSI, seroma, and dehiscence rates	NPWT	Pancreatectomy	Not mentioned	Not mentioned	6–12 mo
O’Neill and Martin^[11]^/USA/RCT	20/20	SSI (3, 7, 15, and 30 d), wound complications	PICO used for 7 d	Hepatic/pancreatic resection	All patients received perioperative antibiotics.	Skin preparation with chlorhexidine	90 d
Lopez-Lopez et al^[18]^/Spain/RCT	54/54	SSI (30 d)	PICO (setting, −80 mm Hg for 5–7 d)	Liver transplant	All patients received.	Not mentioned	30 d
Ceppa et al^[19]^/USA/RCT	75/63	SSI (4, 5, and 30 d)	PREVENA (2–7 d)	Open CRS or open HPB elective surgery	Not mentioned	Not mentioned	30 d
Gupta et al^[20]^/USA/retrospective	25/36	SSI (30 d)	NPWT	Pancreaticoduodenectomy	Perioperative antibiotics	Standard recommendations for SSI reduction were used (wound protector, glove change prior to closure).	30 d
Greene et al^[21]^/Canada/retrospective	61/144	SSI (30 d)	PREVENA (setting, −125 mm Hg for 7 d)	Pancreaticoduodenectomy	Cefazolin and metronidazole were used as the prophylactic antibiotics of choice.	Skin preparation with chlorhexidine	90 d
Mankarious et al^[12]^/USA/retrospective	483/1884	SSI (30 d)	NPWT	Colon resection	Not mentioned	Not mentioned	30 d
Sezikli et al^[22]^/USA/retrospective	47/94	sSSI, dSSI, and seroma	NPWT	High-risk gastrointestinal surgery	All patients received.	Bowel preparation	30 d

CRC = colorectal cancer, CRS = cytoreductive surgery, d = day, dSSI = deep surgical site infection, HPB = hepatobiliary-pancreatic, mm Hg = millimeters of mercury, NPWT = negative pressure wound therapy, PICO = a negative pressure wound therapy system, PREVENA = an incision management system, RCT = randomized controlled trial, SSI = surgical site infection, sSSI = superficial surgical site infection, SWC = surgical wound complication, USA = United States of America.

### 3.3. Quality assessment

Among the 6 RCTs, the risk of bias varied from low to moderate (Figs. 2 and 3^[11,15–19]^). Owing to the physical presence of NPWT devices, blinding of patients, surgeons, and assessors was unfeasible, leading to a high risk of performance bias. Outcome assessment was partially blinded or standardized to alleviate detection bias. All 4 observational studies were evaluated as being of high quality according to the NOS (Table 2).

**Table 2:** Quality assessment of included cohort studies based on the Newcastle-Ottawa Scale (NOS).

Study	Selection				Comparability		Outcome			Total	Rating
	Exposed cohort	Nonexposed cohort	Ascertainment of exposure	Outcome of interest not present at start	Main factor	Additional factor	Assessment of outcome	Follow-up long enough	Adequacy of follow-up		
Gupta et al^[20]^	1	1	1	1	1	0	1	1	1	8	High
Greene et al^[21]^	1	1	1	1	1	1	1	1	1	9	High
Mankarious et al^[12]^	1	1	1	1	1	1	1	1	1	9	High
Sezikli et al^[22]^	1	1	1	1	1	1	1	1	1	9	High

NOS score range: 0 to 9; scores ≥7 indicate high-quality studies, 4 to 6 moderate quality, and ≤3 low quality.

Exposed cohort: patients receiving prophylactic negative pressure wound therapy.

Nonexposed cohort: patients treated with standard surgical dressings.

NOS = Newcastle-Ottawa Scale.

### 3.4. Primary outcome

#### 3.4.1. SSI

**Figure 4.**
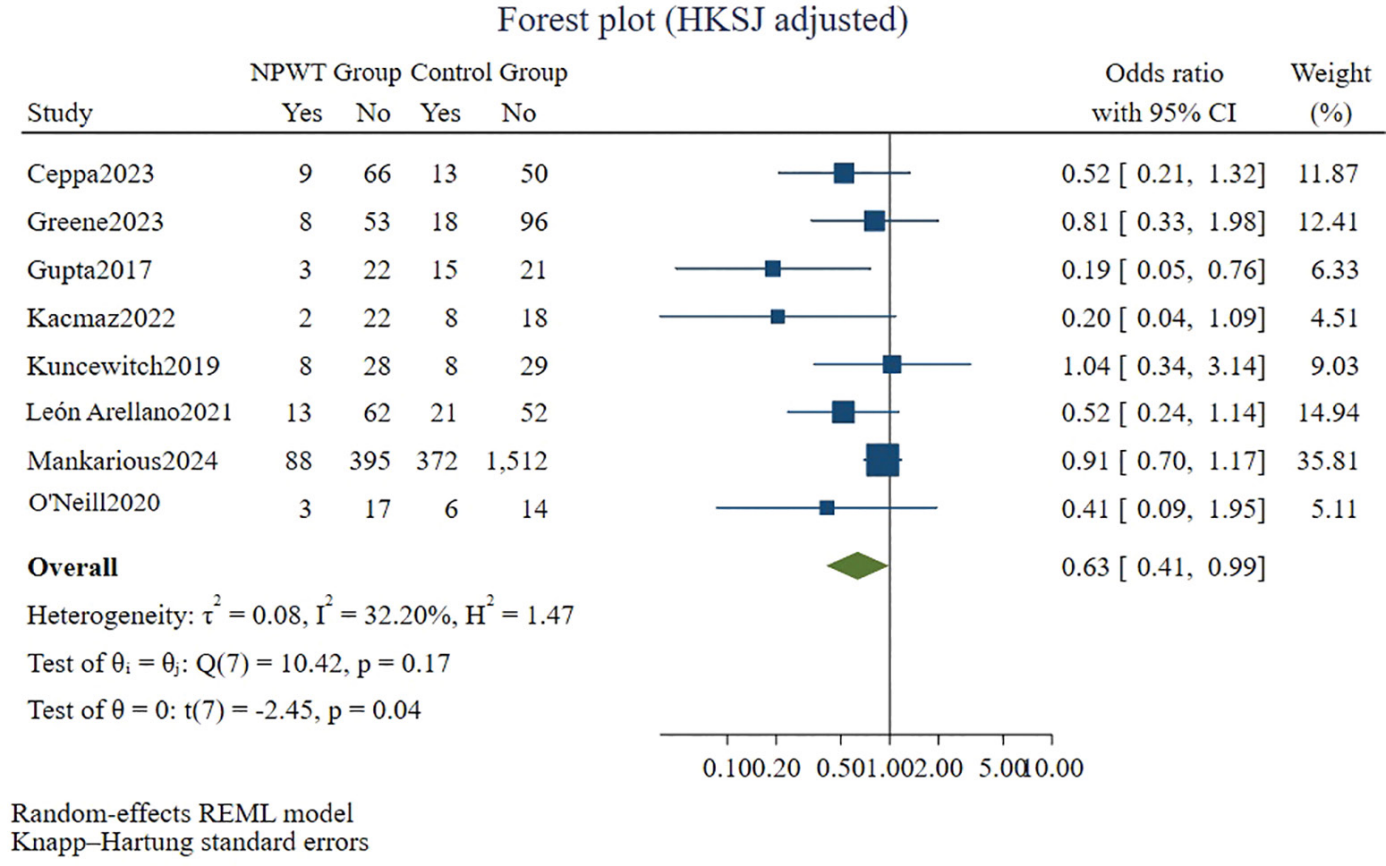
A meta-analysis of NPWT versus conventional dressings on overall SSI rates. CI = confidence interval, HKSJ = Hartung-Knapp-Sidik-Jonkman, NPWT = negative pressure wound therapy, REML = restricted maximum likelihood, SSI = surgical site infection.

Eight studies including 3052 incisions reported overall SSI data.^[11,12,15–17,19–21]^ Using a random-effects REML model with HKSJ adjustment, prophylactic NPWT was associated with a significantly lower risk of the outcome compared with standard dressings (OR = 0.63, 95% CI: 0.41–0.99, *P* = .04). Between-study heterogeneity was low to moderate and not statistically significant (*I*^2^ = 32.2%, τ^2^ = 0.08, *Q* = 10.42, *P* = .17; Fig. 4). These findings suggest that NPWT may reduce the occurrence of this outcome, although the CI was close to the null value and the result should be interpreted with some caution.

### 3.5. Secondary outcomes

#### 3.5.1. Superficial SSI

**Figure 5.**
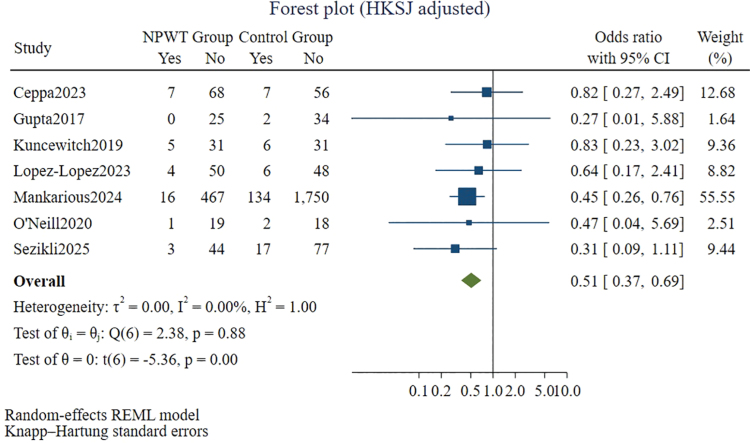
A meta-analysis of NPWT versus conventional dressings on superficial SSI rates. CI = confidence interval, HKSJ = Hartung-Knapp-Sidik-Jonkman, NPWT = negative pressure wound therapy, REML = restricted maximum likelihood, SSI = surgical site infection.

Seven studies including 2928 incisions reported surgical site sSSIs.^[11,12,17–20,22]^ Using a random-effects REML model with HKSJ adjustment, prophylactic NPWT was associated with a significantly lower risk of this outcome compared with standard dressings/control treatment (OR = 0.51, 95% CI: 0.37–0.69, *P* < .01). No statistical heterogeneity was observed among the included studies (*I*^2^ = 0.0%, τ^2^ = 0.00; *Q* = 2.38, *P* = .88; Fig. 5). Most study-specific point estimates favored NPWT, and the pooled estimate indicated a robust protective effect.

#### 3.5.2. Deep SSI

**Figure 6.**
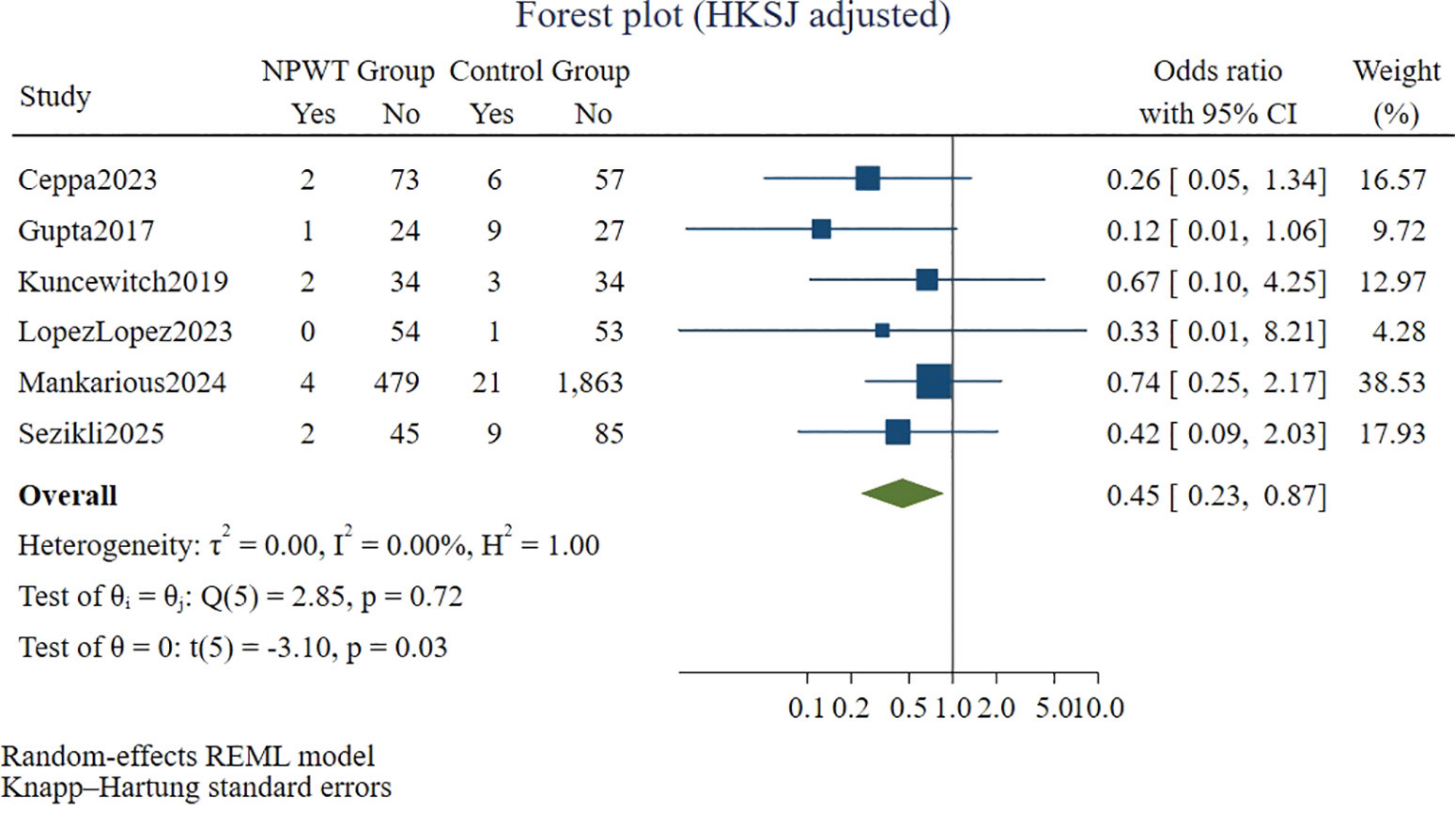
A meta-analysis of NPWT versus conventional dressings on deep SSI rates. CI = confidence interval, HKSJ = Hartung-Knapp-Sidik-Jonkman, NPWT = negative pressure wound therapy, REML = restricted maximum likelihood, SSI = surgical site infection.

Six studies including 2888 incisions reported dSSI.^[12,17–20,22]^ Using a random-effects REML model with HKSJ adjustment, prophylactic NPWT was associated with a significantly lower risk of this outcome compared with standard dressings/control treatment (OR = 0.45, 95% CI: 0.23–0.87, *P* = .03). No statistical heterogeneity was observed among the included studies (*I*^2^ = 0.0%, τ^2^ = 0.00; *Q* = 2.85, *P* = .72; Fig. 6). Although the CIs of individual studies crossed the null line, all point estimates favored NPWT, and the pooled estimate suggested a significant protective effect.

#### 3.5.3. Organ-space SSI

**Figure 7.**
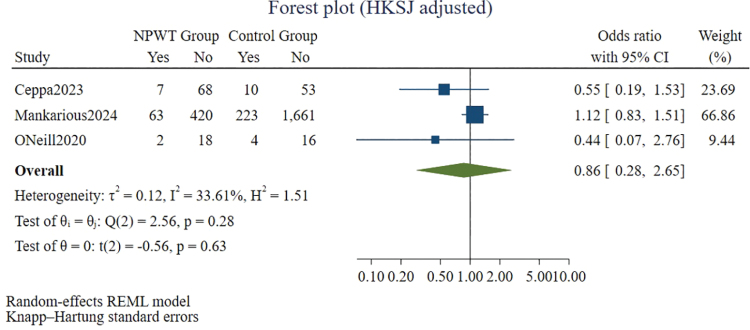
A meta-analysis of NPWT versus conventional dressings on organ-space SSI rates. CI = confidence interval, HKSJ = Hartung-Knapp-Sidik-Jonkman, NPWT = negative pressure wound therapy, REML = restricted maximum likelihood, SSI = surgical site infection.

Three studies including 2545 incisions reported organ-space SSI.^[11,12,19]^ Using a random-effects REML model with HKSJ adjustment, prophylactic NPWT was not associated with a statistically significant reduction in this outcome compared with standard dressings/control treatment (OR = 0.86, 95% CI: 0.28–2.65, *P* = .63; Fig. 7). Low-to-moderate heterogeneity was observed among the included studies, but it was not statistically significant (*I*^2^ = 33.6%, *τ*^2^ = 0.12; *Q* = 2.56, *P* = .28). Given the small number of studies and the wide CI, this result should be interpreted with caution.

#### 3.5.4. Wound dehiscence

**Figure 8.**
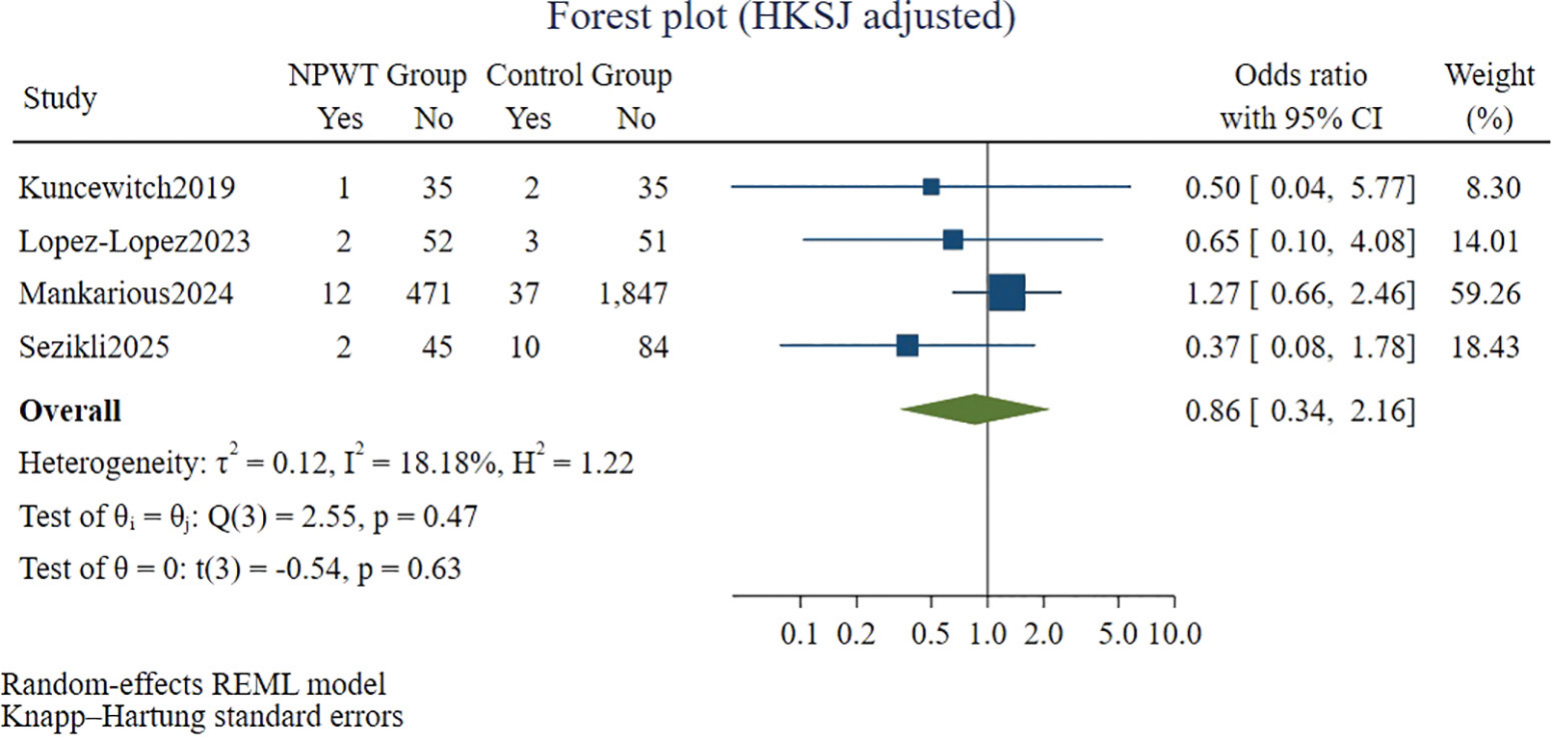
A meta-analysis of NPWT versus conventional dressings on wound dehiscence rates. CI = confidence interval, HKSJ = Hartung-Knapp-Sidik-Jonkman, NPWT = negative pressure wound therapy, REML = restricted maximum likelihood.

Four studies including 2689 incisions reported WD.^[12,17,18,22]^ Using a random-effects REML model with HKSJ adjustment, prophylactic NPWT was not associated with a statistically significant reduction in this outcome compared with standard dressings/control treatment (OR = 0.86, 95% CI: 0.34–2.16, *P* = .63; Fig. 8). Low heterogeneity was observed among the included studies (*I*^2^ = 18.2%, τ^2^ = 0.12; *Q* = 2.55, *P* = .47). Although most study-specific point estimates favored NPWT, the pooled estimate was not statistically significant, and the CI was wide.

### 3.6. Sankey diagram

**Figure 9.**
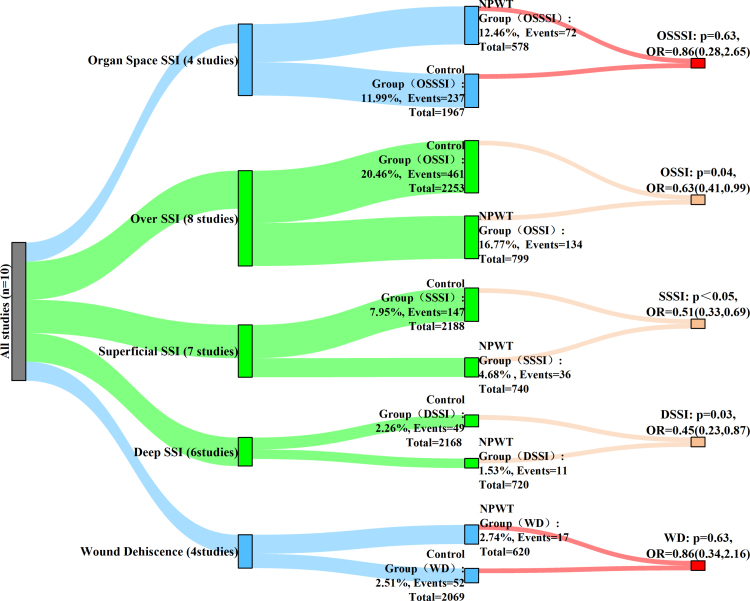
Sankey diagram showing the distribution of included studies, event rates, and pooled effect estimates across surgical site infection outcomes and wound dehiscence. Taupe color: *P* < .05; red color: *P* > .05. dSSI = deep surgical site infection, NPWT = negative pressure wound therapy, OR = odds ratio, OSSI = overall surgical site infection, OSSSI = organ-space surgical site infection, SSI = surgical site infection, sSSI = superficial surgical site infection, WD = wound dehiscence.

A Sankey diagram is a flow diagram that emphasizes the anatomy of connections between categories.^[23]^ To further illustrate the distribution of included studies across surgical categories and outcomes, a Sankey diagram is shown in Figure 9. Among the 10 included studies, 8 reported overall SSI, 7 reported sSSI, 6 reported dSSI, 4 reported organ-space SSI, and 4 reported WD. The Sankey diagram showed that the event rates were generally lower in the NPWT group than in the control group for overall SSI, sSSI, and dSSI, consistent with the pooled meta-analysis results. Specifically, NPWT was associated with a significant reduction in overall SSI (OR = 0.63, 95% CI: 0.41–0.99, *P* = .04), sSSI (OR = 0.51, 95% CI: 0.37–0.69, *P* < .001), and dSSI (OR = 0.45, 95% CI: 0.23–0.87, *P* = .03). In contrast, no significant differences were observed for organ-space SSI (OR = 0.86, 95% CI: 0.28–2.65, *P* = .63) or WD (OR = 0.86, 95% CI: 0.34–2.16, *P* = .63).

### 3.7. Sensitivity analyses

Leave-one-out sensitivity analyses were performed to evaluate the influence of individual studies on the pooled estimates. For the overall SSI outcome (Fig. S1, Supplemental Digital Content 1), the pooled effect estimates remained consistently in favor of NPWT after sequential exclusion of each study, although statistical significance was attenuated in several analyses, suggesting moderate robustness. For the sSSI outcome (Fig. S2, Supplemental Digital Content 3), the pooled estimates remained statistically significant after omission of any single study, indicating a robust protective effect of NPWT. For the dSSI outcome (Fig. S3, Supplemental Digital Content 4), the direction of effect remained unchanged after each study was omitted, but statistical significance was not consistently maintained. For the organ-space SSI and WD outcomes (Figs. S4 and S5, Supplemental Digital Content 5), the pooled estimates remained nonsignificant in all leave-one-out analyses, with wide CIs, suggesting limited precision and insufficient evidence for a significant effect. Overall, the sensitivity analyses indicated that the beneficial effects of NPWT were generally directionally consistent, but the robustness varied across outcomes, particularly for outcomes with fewer studies and wider CIs (Table S1, Supplemental Digital Content 2).

## 4. Discussion

SSI remains a significant complication in abdominal and GI surgery. NPWT has emerged as a promising strategy for SSI prevention in diverse surgical domains. Nevertheless, the universal applicability of NPWT remains a subject of debate. For instance, in patients undergoing GI surgery, Ceppa et al^[19]^ reported no statistically significant difference between NPWT and standard dressings in reducing incisional SSI (14% vs 17%, *P* = .31) or organ-space SSI (11% vs 13%, *P* = .35). Similarly, in complex pancreatic surgeries, Andrianello et al^[24]^ and Moreno Gijón et al^[25]^ found no significant differences in overall SSI rates between NPWT and conventional dressings. These findings imply that the benefits of NPWT may be contingent upon the type of surgery, the surgical site, and the patient’s baseline conditions and are not universally applicable to all clean-contaminated abdominal incisions.

This review emphasizes the evidence supporting the application of NPWT in closed abdominal incisions following GI and HBP surgery. Findings from multiple prospective studies and meta-analyses suggest that NPWT significantly reduces overall SSI rates among high-risk patients. Stratified analyses show that the reduction in sSSI primarily reflects the decline in skin and subcutaneous tissue infections, whereas the reduction in dSSI is related to the fascial and muscular layers. NPWT offers multiple mechanistic advantages, such as the redistribution of lateral tension to enhance mechanical stability and the disruption of pathophysiological processes underlying superficial and mid-layer infections. Wilkes et al^[26]^ demonstrated that NPWT can reduce lateral stress around the incision by approximately 50% and redirect stress distribution. Animal studies imply that NPWT enhances lymphatic clearance from the subcutaneous dead space, thus reducing the formation of hematomas and seromas.^[27]^

At the molecular level, NPWT reduces the local concentrations of pro-inflammatory cytokines, such as tumor necrosis factor-α and interleukin-1β, while enhancing the levels of pro-angiogenic factors, including interleukin-8 and vascular endothelial growth factor.^[28,29]^ This promotes an anti-inflammatory and pro-angiogenic microenvironment conducive to wound healing. Nevertheless, NPWT does not exert a significant impact on organ-space SSI or complete WD. This is presumably due to multifactorial influences, such as surgical techniques, anastomotic integrity, patient conditions, and abdominal contamination, which are beyond the scope of local incision interventions.

The quality assessment using the Risk of Bias Tool 2.0 and NOS tools indicated that the included studies were generally methodologically robust. Given the limited number of studies available for several outcomes, we applied a random-effects model with HKSJ adjustment to obtain more conservative estimates. The leave-one-out sensitivity analyses generally supported the robustness of the main findings; however, some secondary outcomes appeared to be influenced by single high-weight studies, as reflected by wider CIs after their exclusion. In addition, the Sankey diagram showed that the available evidence was unevenly distributed across outcomes, with more studies reporting overall and sSSI than organ-space SSI or WD. This imbalance may partly explain the imprecise and nonsignificant estimates for these less frequently reported outcomes. Therefore, although our findings support a protective effect of NPWT against overall, superficial, and dSSI, the results for secondary and less frequently reported outcomes should be interpreted cautiously. Several additional limitations should also be acknowledged, including potential performance and detection biases related to the unblinded nature of NPWT devices, as well as heterogeneity in study design, sample size, follow-up duration, and device type. Some included studies may also have been underpowered because of small sample sizes. Moreover, cost-effectiveness remains an important consideration. Although NPWT may reduce SSI-related morbidity, its relatively high upfront cost may limit routine use in low-risk surgeries, as noted by Flynn et al^[30]^ and Ceppa et al.^[19]^ Further adequately powered studies are warranted to validate these findings and better define the clinical and economic contexts in which NPWT is most beneficial.

## 5. Conclusion

NPWT reduces the rates of SSI, sSSI, and dSSI among high-risk patients undergoing abdominal surgery, whereas organ-space SSI and WD remain unaffected. Future research ought to concentrate on the standardization of NPWT protocols, cost-effectiveness analyses, and the identification of patient populations most likely to benefit.

## Acknowledgments

I would like to express my sincere gratitude to my professor, Chao Liu. His expertise and guidance were instrumental in enhancing the quality of the content. I am truly thankful for his generosity in sharing his experience.

## Author contributions

**Conceptualization:** Yanyun Long.

**Data curation:** Yanyun Long.

**Formal analysis:** Yanyun Long.

**Investigation:** Yanyun Long, Kelei Wang, Yong Guan.

**Methodology:** Yanyun Long.

**Project administration:** Chao Liu.

**Resources:** Xiaohui Gu.

**Supervision:** Chao Liu.

**Software:** Yanyun Long, Xin Wang, Kelei Wang, Shaocong Wang.

**Validation:** Chao Liu.

**Writing – original draft:** Yanyun Long.

**Writing – review & editing:** Yanyun Long.












