# Supplementary material for: Prophylactic negative pressure wound therapy reduces surgical site infection in gastrointestinal, hepatobiliary, and pancreatic laparotomy: A systematic review and meta-analysis
Source: Medicine (Baltimore). 2026 Aug 14;105(33):e50174. doi: 10.1097/MD.0000000000050174 (PMC13480752; doi:10.1097/MD.0000000000050174)
Supplement: Supplementary file 1 [file medi-105-e50174-s002.doc]

| **Figure** | **Overall Pooled Estimate** | **OR Range After**  **Leave-One-Out Analysis** | **Direction Maintained**  **After Omission** | **Statistical Significance Maintained After Omission** | **Robustness Assessment** | **Interpretation** |
| --- | --- | --- | --- | --- | --- | --- |
| Overall SSI | 0.63  (0.41–0.99) | 0.53–0.73 | Yes, all estimates remained <1 | No, several CIs crossed or approached 1 | Moderate to weak | The direction of effect was consistent, but statistical significance was sensitive to the omission of some studies. |
| Superficial SSI | 0.51  (0.37–0.69) | 0.47–0.59 | Yes, all estimates remained <1 | Yes, all CIs remained below 1 | Strong | The pooled result was robust and was not materially influenced by any single study. |
| Deep SSI | 0.45  (0.23–0.87) | 0.33–0.52 | Yes, all estimates remained <1 | No, some CIs crossed 1 | Moderate | The direction of effect was stable, but statistical significance was not consistently maintained. |

Supplementary Table S1. Summary of leave-one-out sensitivity analyses

Supplementary Table S1 (continued)

| **Figure** | **Overall Pooled Estimate** | **OR Range After**  **Leave-One-Out Analysis** | **Direction Maintained**  **After Omission** | **Statistical Significance Maintained After Omission** | **Robustness Assessment** | **Interpretation** |
| --- | --- | --- | --- | --- | --- | --- |
| Organ-space SSI | 0.86  (0.28–2.65) | 0.52–1.09 | No, the estimate exceeded 1 after omission of Ceppa2023 | No, all CIs crossed 1 | Weak | The result remained non-significant, with wide CIs indicating limited precision and insufficient evidence for a clear effect. |
| Wound dehiscence | 0.86  (0.34–2.16) | 0.48–1.12 | No, the estimate exceeded 1 after omission of Sezikli2025 | No, all CIs crossed 1 | Weak/unstable | The pooled estimate was influenced by some individual studies and did not show a clear protective effect. |

**Abbreviations:** CI, confidence interval; OR, odds ratio; SSI, surgical site infection.

**Notes:** Direction maintenance indicates whether the pooled effect estimate remained on the same side of the null value after sequential omission of each study. Statistical significance was considered maintained when the 95% CI did not cross 1 in all leave-one-out analyses.
